# The Wassermann Reaction in the Diagnosis of Syphilis

**Published:** 1912-04

**Authors:** J. Edgar Paullin

**Affiliations:** Atlanta, Ga.


### Journal-Record of Medicine

Successor to Atlanta Medical and Surgical Journal, Established 1855, and Southern Medical Record, Established 1870.

OWNED BY THE ATLANTA MEDICAL JOURNAL CO.

Published Monthly

*Official Organ Fulton County Medical Society, State Examining Board, Presbyterian Hospital, Atlanta, Birmingham and Atlantic Railroad Surgeons' Association, Chattahoochee Valley Medical and Surgical Association, Etc.*

EDGAR G. BALLENGER, M. D., Editor.
BERNARD WOLFF, M. D., Supervising Editor.
A. W. STIRLING, M. D., C. M., D. P. H., J. S. HURT, B. Ph., M. D.
GEO. M. NILES, M. D., W. J. LOVE, M. D., (Ala.); Associate Editors.
E. W. ALLEN, Business Manager.

COLLABORATORS

Dr. W. F. WESTMORLAND, General Surgery.
F. W. McRAE, M. D., Abdominal Surgery.
H. F. HARRIS, M. D., Pathology and Bacteriology.
E. B. BLOCK, M. D., Diseases of the Nervous System.
MICHAEL HOKE, M. D., Orthopedic Surgery.
CYRUS W. STRICKLER, M. D., Legal Medicine and Medical Legislation.
E. C. DAVIS, A. B., M. D., Obstetrics.
E. G. JONES, A. B., M. D., Gynecology.
R. T. DORSEY, Jr., B. S. M. D., Medicine.
L. M. GAINES, A. B., M. D., Internal Medicine.
GEO. C. MIZELL, M. D., Diseases of the Stomach and Intestines.
L. B. CLARKE, M. D., Pediatrics.
EDGAR PAULIN, M. D., Opsonic Medicine.
THEODORE TOEPEL, M. D., Mechano Therapy.
R. R. DALY, M. D., Medical Society.
A. W. STIRLING, M. D., etc., Diseases of the Eye, Ear, Nose and Throat.
BERNARD WOLFF, M. D., Diseases of the Skin.
E. G. BALLENGER, M. D., Diseases of the Genito-Urinary Organs.

VOL. LIX. APRIL 1912 NO. 1

### THE WASSERMANN REACTION IN THE DIAGNOSIS OF SYPHILIS.

J. EDGAR PAULLIN, ARLANTA, GA.

For a complete understanding of the Wassermann reaction it is necessary to briefly refer to certain fundamental facts commonly manifest in the study of immunity. These facts are but the principles enunciated by Ehrlich in his so-called "side-chain" theory.

When an animal is inoculated or becomes infected with a proteid substance foreign to its body, there develops in the blood of this animal certain substances which are antagonistic to the foreign protein. These antagonistic substances are commonly known as "anti-bodies." On the other hand, the foreign protein which gains entrance into the body and gives rise to the production of anti-bodies, is known as "antigen." Antibodies are either simple or complex; they may be such substances as antitoxins, agglutinins, lysins, precipitins, opsonins, etc. The simple type of anti-body is well illustrated in diphtheria antitoxin —whereas the more complex type known as "amboceptor" in the present study, is the one which interests us, and it might be remarked that an amboceptor is an anti-body which can act antagonistically against its particular antigen only by means of the presence of another subject known as "complement."

To illustrate the reaction which occurs in the case of an amboceptor, we will make use of the following experiments: in a test tube we mix a certain amount of rabbit serum with human red blood cells which have been washed free from plasma. Put the mixture in an incubator for one hour. At the end of this time no change will be observed in the corpuscles. On the other hand, if this rabbit has received intraperitoneally or intravenously five or six injections of washed human red cells, given at five or six day intervals, and each dose administered has been larger than the previous one, it will be found that the serum of this rabbit when mixed with washed human red cells, incubated for one hour at 37° C., the mixture then examined, the red cells will have disappeared, and the fluid will have a clear, transparent, reddish color. The rabbit's serum has developed an entirely new property on account of the injection of human red cells, and the reaction just stated (haemolysis), is due to the presence in the rabbit's serum of an entirely new substance—amboceptor—specific for human red cells. By careful titration, it will be found that the rabbit's serum can be greatly diluted and at the same time possess this haemolytic substance. If this rabbit's serum is heated to 55 degrees C. for thirty minutes, and then mixed as in the previous experiment with human red cells, it will be found that no dissolution of the corpuscles takes place. This is occasioned by the fact that the complement which is normally present in the rabbit's serum has been destroyed by heating. If we add to this same mixture one or two drops of guinea pig's serum, the reaction will again occur with as much intensity as formerly observed. By adding the guinea pig's serum we have supplied the complement (previously destroyed by heating) which is necessary for the reaction. The complement completing the union of red-blood-cell amboceptor combination. Heating to 55 degrees C. for thirty minutes does not affect the amboceptor.

The above mentioned reaction is a specific reaction, because the blood serum of this particular rabbit will dissolve or haemolyze the red corpuscles of man, and no other animal. In other words, if the rabbit's serum is mixed with the red corpuscles of the sheep, goat, dog, cat, etc., the cells after incubation will remain unchanged.

The same principle holds true regarding bacteria. By injecting a rabbit with frequent and large doses of typhoid bacilli, the rabbit will respond to these injections by developing in his blood an amboceptor which, when mixed outside of the body with typhoid bacilli in the presence of complement, will accomplish a rapid destruction of these organisms.

Thus far we have only considered experimental work, although the same principle holds true in most infectious diseases. For example, when an individual becomes infected with syphilis or with trepanoma pallida, the organism of syphilis, in the great majority of cases the body at once begins to respond to this infection just as the rabbit responds to the injection of red blood cells, though the response is not as intense. The antibodies thrown into the circulation by the entrance of this organism are amboceptors, and the Wassermann reaction is an attempt to detect in the blood of an individual having this disease, the presence of these specific anti-bodies.

In the present state of our knowledge it is not possible to determine either microscopically or macroscopically the reaction which occurs when syphilitic antigen, syphilitic serum and complement are brought together. A change does take place in the mixture, the three uniting, but in order to discover this union it is necessary to add an anti-human amboceptor which has been inactivated by heating to 55 degrees C. for thirty minutes (destroying the complement), and the human red cells, and again incubate. Having added a known amount of complement to the previous syphilitic mixture, it is easy to show that union has occurred between the antigen, syphilitic serum, and complement, because there is no excess of complement to unite with the human red cells and antihuman amboceptor to cause haemolysis. The mixture, therefore, remains the same after this last incubation. Should we use a serum from an individual who hasn't syphilis, there will then be no syphilitic antibodies in this serum to unite with the antigen and complement, consequently when the atihuman amboceptor and red cells are added, dissolution of red cells or haemolysis takes place, because the complement is free in the mixture and therefore unites with the latter combination.

So much for the condensed resume of the preliminary steps of the reaction. Concerning the reaction itself the reagents necessary for the performance of the test are:

(1) Suspected Serum.
(2) Complement.
(3) Antigen.
(4) Washed Human Red Cells.
(5). Anti-human Amboceptor.

(1) *Suscepted Serum.*—The blood for examination is generally obtained by inserting the needle of an hypodermic syringe into the median vein of the arm, and withdrawing at least 2 or 3 cc. of blood. The suscepted blood is at once put into a sterile bottle, properly corked, and allowed to coagulate. In some individuals it is very easy to obtain the blood necessary for the reaction from the tip of the finger or the lobe of the ear. In this event, the blood is collected in a capillary tube, having a capacity of at least 2 cc. Unless one is skilled in the use of the tube the blood will coagulate before a sufficient quantity is collected, and on this account the removal of blood from the vein is more desirable. After coagulation the clear serum is withdrawn by means of a capillary pipette, heated in a water-bath to 55 degrees C. in order to destroy whatever complement that should happen to be present.

(2) *Complement.*—It has been determined by numerous experiments that the complement normally present in the blood of human beings and of rabbits is quite variable. It was found out that guinea pig's serum contained very nearly a uniform amount of complement, and for this reason the serum of this animal is used in the test. The blood is obtained from the carotid artery, or a sterile hypodermic needle is inserted directly into the heart and two or three cc. of blood removed. After coagulation the serum is diluted, titrated, and is then ready for use. The serum unless kept at 0 degrees C. does not long retain the complement, so that it is usually necessary to always work with a fresh specimen.

(3) *Antigen.*—On account of the difficulty which has been experienced in growing artificially the trepanoma pallida, it has been impossible to have an antigen prepared directly from the specific organism causing syphilis. As a substitute for this, an alcoholic extract of a syphilitic foetal liver, which contains spirochaetes in abundance is used. By experimentation it has been demonstrated that even the use of an extract from a syphilitic foetal liver is not necessary, and in its place one can use the extract made from beef's heart, liver, or guinea pig's heart or liver. Because of this fact, that the antigen necessary in this reaction is not of necessity syphilitic in nature, the test has been greatly criticised, although the criticism has by no means weakened the clinical value of the reaction. Not knowing the nature of the syphilitic antigen, we feel certain from the fact that an antigen made from the heart, kidney or liver, which has been extracted with alcohol, the alcohol evaporated, and the residue taken up with a small quantity of ether, again precipitated from this etherial solution by the addition of five volumes of acetone, the precipitate collected, dried, and dissolved in methyl alcohol furnishes an antigen, lipoid in character, which does unite with the syphilitic anti-body, and serves well for this purpose in the reaction. Clinical results more than confirm this. The antigen thus prepared is diluted with 0.9 per cent. saline solution and titrated against a known syphilitic serum to establish its strength. Once determined the titre for this preparation will remain fairly constant.

(4) *Washed Human Red Cells.*—About 1-2 cc. of blood from an individual who gives a negative Wassermann is collected from the finger or lobe of the ear; this is twice washed by centrifugation in 0.9% sodium chloride solution to free the cells from plasma. After washing the cells are diluted with normal saline until a 5% mixture is made.

(5.) *Anti-human Amboceptor.*—In the original Wassermann technique an anti-sheep amboceptor is used, which was prepared by injecting rabbits with sheep corpuscles,—this necessitated in the reaction the use of sheep's corpuscles. Noguchi called attention to certain disadvantages in using sheep's corpuscles with human serum in the test, and suggested the use of the anti-human system. After doing parallel tests on one hundred different specimens of blood and observing no difference whatever in the end reaction in any case, because of the ease with which human cells can be obtained, the anti-human system is employed. This amboceptor is prepared by the intravenous or intraperitoneal injection at intervals of increasing doses of human red blood cells into rabbits as previously described. After seven or eight injections, the rabbit is bled under aseptic conditions and the serum inactivated and tested to determine its strength.

*The Performance of the Test* consists in mixing varying amounts (½, ¼, ⅛ cc. of 25% dilution), of suspected serum, a known amount of complement, and a known amount of antigen,—the complement and antigen strengths having been previously determined by titration against a known syphilitic serum—the mixture incubated for one hour at 37 degrees C. or still better, placed in a water-bath at this temperature. After this incubation 1 cc. of the red cell dilution and two units of amboceptor are added to each tube; again incubate with frequent shaking for two hours, or until haemolysis is complete in the control tubes; the end reaction can then be studied. Control tests are made of each re-agent entering the mixture to be certain they do not cause or prevent haemolysis. With each set of tests there is always used two control serums—one of these known to give a positive Wassermann and one from an individual known to give a negative test.

*The interpretation of the result* after having employed the most careful technique is at times as difficult as the performance of the test. Most clinicians think that a blood tested comes through either positive or negative, on the other hand, there are occasionally bloods which give various degrees of a reaction, the degree being expressed in the percentage of haemolysis occurring in the tubes, the question naturally arises as to how one should interpret the intermediate reactions. Occasionally in making the test the tube containing ½ cc. of the serum will show a parial inhibition of haemolysis, the tube containing ¼ cc. a slighter amount and that containing ⅛ cc. no inhibition. In such a serum the amount of reacting substance is slight, and as to whether the individual in thise case should be reported positive or negative depends on—whether the serum has reacted positively, or whether the individual gives clinically a definite syphilitic history. If either of these are true then the reaction is called positive. If a serum tested for the first time and with no further evidence of syphilis than this partial reaction one is not justified in making a diagnosis of syphilis from this amount of a reaction. However, if the patient has been under anti-syphilitic treatment and the blood shows this much of a reaction even though the treatment has been administered over a long period of time, one is justified in making a positive diagnosis from this reaction. Without other evidences of syphilis than the partial reaction, a positive diagnosis based on this would frequently be wrong, because of a certain lack of specificity in the reaction. Therefore, a positive test is only reported as such when there is a complete inhibition of haemolysis in all the tubes or a very decided difference in the color of the haemoglobin, with marked precipitation of the red cells in the antigen containing tubes.

From June, 1909, to February, 1912, I have made twelve hundred and seventy Wassermann tests; in the great majority of these, eight hundred and twenty-one, the original technique of Wassermann was carried out, in the remainder the technique is the same, but the anti-human system was substituted for the anti-sheep system. Of this number, one hundred and seventy-three tests were on the blood of negroes, with the result that ninety-six were positive and seventy-seven negative. Of the ten hundred and ninety-seven whites, four hundred and eighty-two reacted positively and six hundred and fifteen negatively.

The twelve hundred and seventy tests were done on the bloods of nine hundred and eighty-two different individuals; some having had as many as five examinations at different times. Of the nine hundred and eighty-two cases, I have clinical data concerning four hundred and twenty-five patients, covering six hundred and nine Wassermann's. In a great majority of these cases the clinical data is exceedingly meager, so much so, that I am unable to make a definite classification as to the various stages of the disease at which the tests were made,—on this account it is practically impossible to group the cases into the primary, secondary, and tertiary stages, as is usually done. Of the number four hundred and twenty-five, one hundred and seventy-nine cases reacted positively, two hundred and forty-six negatively.

There are one hundred and thirteen cases showing an initial lesion; sixty-two reacted positively, and fifty-one negatively. Of the latter number nineteen subsequently became positive before the appearance of the rash, while thirty-two up to the present time have manifested no further evidence of syphilis.

There are seventy-eight cases giving a history of primary lesion, fifty-three of these having had secondaries, treated for several years with mercury and potash, exhibiting at the time no clinical evidence of syphilis, with a negative Wassermann. Belonging to this same class there were twenty-two cases giving a positive reaction, which subsequently cleared up under the use of salvarsan.

There are twenty-seven cases giving a history of having had syphilis and having taken active treatment from two to four years, showing at the time of test suspicious evidence of the disease, twelve reacted positively, and fifteen negatively; six of the negatives subsequently gave a decidedly positive test from two to four days after the administration of salvarsan.

The bloods of thirty-one adults with no history of syphilis were examined for diagnostic purpose. Of this number twenty-nine reacted negatively and two positively. The two positive cases were undoubtedly cases of congenital lues. One of these individuals had had as a child an osteomyelitis of the lower jaw, the humerus of the left arm, the right femur, and the right tibia. At the time I examined him he was suffering with a chronic nasal catarrh, the liver was enlarged, and the lymph glands were palpatable. After three doses of 606, liver returned to its normal size, and the trouble of which he complained at the time disappeared.

Twelve children under fourteen years of age, suspected of having congenital lues were examined. Of this number five reacted positively, and it is an interesting fact that three of the mothers of these children gave a positive reaction, although giving no clinical evidence of infection.

Fifty-nine cases have been examined after having received two or three doses of salvarsan, the interval of time between the last injection of salvarsan and the Wassermann test varied from one to six months; all negative. In fourteen cases from one to three months after one dose of salvarsan, six gave positive reactions.

I have examined the blood of fifteen cases of tabes dorsalis, of this number six gave a positive Wassermann. Ten of the fifteen received intravenous doses of salvarsan, and the only ones benefited by this treatment were those giving a positive reaction; the negative cases had been treated for a considerable length of time with potash and mercury.

Of particular interest is a case with a gumma of the frontal bone present for two or three years; this patient had his primary infection eighteen years previously, treated at that time with mercury for three years. Wassermann positive,—condition cleared up with salvarsan.

Three cases of undoubted syphilitic infection of the lung which presented all the physical signs of tuberculosis, but being unable to find tubercle bacilli in any of the specimens of sputum, a Wassermann was made, each giving a positive reaction; after the administration of anti-syphilitic treatment, the physical signs immediately disappeared.

Two very interesting cases of syphilitic nephritis have been under my obsevation. In each case the urine showed a considerable trace of albumen and any number of hyaline and granular cases in several specimens examined. On account of the positive Wassermann test they were given anti-syphilitic treatment, with a prompt disappearance of the albumen and casts in the urine.

I have had two cases under observation to determine the length of time it would take mercury to make a positive Wassermann become negative. Both patients were given ¼ gr. protoiodide of mercury three times a day for one week, an examination of their bloods at the end of this time gave 75% positive. After two weeks of mercury the reaction had disappeared. Mercury was then omitted and the reaction again became positive in eighteen days. A positive Wassermann can be made negative by the hypodermic administration of mercury. The administration of iodide of potash in large dosese causes a positive Wassermann to become negative, although not as rapidly as mercury.

There still remains five hundred and seven cases covering six hundred and sixty Wassermann tests, with no data to cover the clinical history. The greater number of these tests were made on bloods sent me by physicians in this and other States for examination, and I have been unable to obtain information concerning these patients.

Out of the five hundred and seventy-eight positive reactions, the test was only questioned in one case, so far as I have been able to find out—that of a man who had been for years a large consumer of morphine, the reaction on this blood was positive. As to whether he had syphilis or the reaction was affected by the morphia, it is not possible to say.

It might be stated that a positive Wassermann test undoubtedly means that the individual has syphilis. The only other diseases in which a positive reaction is obtained are, rarely in the acute stage of scarlet fever, occasionally in leprosy, and in yaws. It is also quite true that occasionally a negative reaction is obtained on the blood of a patient who clinically has syphilis; at the same time, if this individual is closely questioned, it will be found that he has recently taken either mercury or potash.

In the primary and secondary manifestations of the disease the reaction is of a great deal of value in determining the nature of the infection. The negative reaction under these circumstances makes syphilis very improbable.

The Wassermann reaction is of untold value in judging of the cure of an individual who has once had syphilis. After thorough medication the test should be made three, six and twelve months after the cessation of treatment. Should the individual not be cured the blood will give a positive reaction a considerable length of time before there is any clinical evidence of the disease.

Suite 612 Peters Building.

